# Beyond the Acute Phase: Long-Term Impact of COVID-19 on Functional Capacity and Prothrombotic Risk—A Pilot Study

**DOI:** 10.3390/medicina60010051

**Published:** 2023-12-27

**Authors:** Doina-Clementina Cojocaru, Florin Mitu, Maria-Magdalena Leon, Lucia Corina Dima-Cozma, Cristina Andreea Adam, Carmen Marinela Cumpăt, Robert D. Negru, Alexandra Maștaleru, Viviana Onofrei

**Affiliations:** 1 Department of Medical Specialties I and III, “Grigore T. Popa” University of Medicine and Pharmacy, University Street No. 16, 700115 Iasi, Romania; 2 Clinical Rehabilitation Hospital, Cardiovascular and Respiratory Rehabilitation Clinic, Pantelimon Halipa Street No. 14, 700661 Iasi, Romania; 3 Academy of Medical Sciences, 030167 Bucharest, Romania; 4 Academy of Romanian Scientists, 700050 Iasi, Romania; 5 “St. Spiridon” Clinical Emergency Hospital, Cardiology Department Independence Boulevard No. 1, 700111 Iasi, Romania

**Keywords:** long COVID, body plethysmography, functional capacity, prothrombotic risk, D-dimers, COVID-19, DLCO

## Abstract

*Background and Objectives*: Assessment of the prothrombotic, proinflammatory, and functional status of a cohort of COVID-19 patients at least two years after the acute infection to identify parameters with potential therapeutic and prognostic value. *Materials and Methods*: We conducted a retrospective, descriptive study that included 117 consecutive patients admitted to Iasi Pulmonary Rehabilitation Clinic for reassessment and a rehabilitation program at least two years after a COVID-19 infection. The cohort was divided into two groups based on the presence (*n* = 49) or absence (*n* = 68) of pulmonary fibrosis, documented through high-resolution computer tomography. *Results*: The cohort comprises 117 patients, 69.23% females, with a mean age of 65.74 ± 10.19 years and abnormal body mass index (31.42 ± 5.71 kg/m^2^). Patients with pulmonary fibrosis have significantly higher levels of C-reactive protein (CRP) (*p* < 0.05), WBC (7.45 ± 7.86/mm^3^ vs. 9.18 ± 17.24/mm^3^, *p* = 0.053), neutrophils (4.68 ± 7.88/mm^3^ vs. 9.07 ± 17.44/mm^3^, *p* < 0.05), mean platelet volume (MPV) (7.22 ± 0.93 vs. 10.25 ± 0.86 fL, *p* < 0.05), lactate dehydrogenase (*p* < 0.05), and D-dimers (*p* < 0.05), but not ferritin (*p* = 0.470), reflecting the chronic proinflammatory and prothrombotic status. Additionally, patients with associated pulmonary fibrosis had a higher mean heart rate (*p* < 0.05) and corrected QT interval (*p* < 0.05). D-dimers were strongly and negatively correlated with diffusion capacity corrected for hemoglobin (DLCO corr), and ROC analysis showed that the persistence of high D-dimers values is a predictor for low DLCO values (ROC analysis: area under the curve of 0.772, *p* < 0.001). The results of pulmonary function tests (spirometry, body plethysmography) and the 6-minute walk test demonstrated no significant difference between groups, without notable impairment within either group. *Conclusions:* Patients with COVID-19-related pulmonary fibrosis have a persistent long-term proinflammatory, prothrombotic status, despite the functional recovery. The persistence of elevated D-dimer levels could emerge as a predictive factor associated with impaired DLCO.

## 1. Introduction

Since December 2019, when cases of pneumonia of unknown etiology were first reported in Wuhan (China) [1], until recently, about 600 million people have been diagnosed with coronavirus disease 2019 (COVID-19), and about 6.5 million have died secondary to this infection. Epidemiological data with a strong medical, social, and economic impact have outlined a real public health problem [2]. 

Long COVID, also known as Post-Acute Sequelae of COVID-19 has been described as a pathology with potential multisystem involvement, in the context of persistence of symptoms at least 4 weeks after the acute infectious stage, having an undulating character, with periods of exacerbation and remission that may persist for a variable period of time up to years. To date, cases have been reported both in patients with severe forms of COVID-19 (50–70% of them) and in those with pauci-symptomatic forms [3,4]. Myocardial inflammation, cardiac dysfunction, and endothelial dysfunction (which promotes platelet adhesion and coagulation leading to microangiopathies and a prothrombotic status) are the main findings reported at the cardiovascular level in these patients [5,6,7]. Of all COVID-19 secondary respiratory complications, pulmonary fibrosis is accompanied by a high morbidity and according to the limited epidemiological data available to date, the prevalence is about 7% [8]. According to existing data in the literature, the understanding of the pathophysiological mechanisms is not fully clarified, an aspect that modulates both the treatment and prognosis of patients evaluated at a distance from the acute episode of COVID-19 [9].

Clinical studies conducted recently have identified several predictors for the development of pulmonary fibrosis in known patients with COVID-19 infection, such as female gender, older age, a high number of comorbidities, and the need for mechanical ventilation during the acute episode [9]. Moreover, high serum levels of C-reactive protein (CRP) or D-dimer are accompanied by a high risk of maintaining pulmonary functional abnormalities [10]. In patients with COVID-19 infection, the persistence of a systemic inflammatory status has been shown to induce an autoimmune response with excess production of cytokines and autoantibodies, which may contribute to both the development and progression of parenchymal lung lesions in this category of patients [11,12,13]. In addition to the risk factors presented above, genetic studies have shown that a decrease in leukocyte telomeres can contribute to developing pulmonary fibrosis [14].

COVID-19 is characterized by an accentuated proinflammatory status associated with endothelial dysfunction and persistent prothrombotic status (thrombosis of the lung microvascular circulation and arterial thrombosis) due to persistent chronic infection, hypoxia, and inflammatory response [15,16,17,18,19]. Recently, three possible explanations for the concept of *thromboinflammation* along COVID-19 have been proposed: the persistence of structural changes (especially endothelial dysfunction secondary to acute infection), the persistence of a viral reservoir, and associated immunological disturbances [20]. D-dimer assessment is recommended to be performed periodically in patients with long COVID, as this biological parameter is a known risk factor associated with thromboembolic complications [21]. 

Our study aimed to evaluate the proinflammatory, prothrombotic, and functional status of a cohort of COVID-19 patients long term (at least two years) after acute infection to identify several biological and functional parameters with potential therapeutic and prognostic impacts.

## 2. Materials and Methods

### 2.1. Study Design

**Figure 1:**
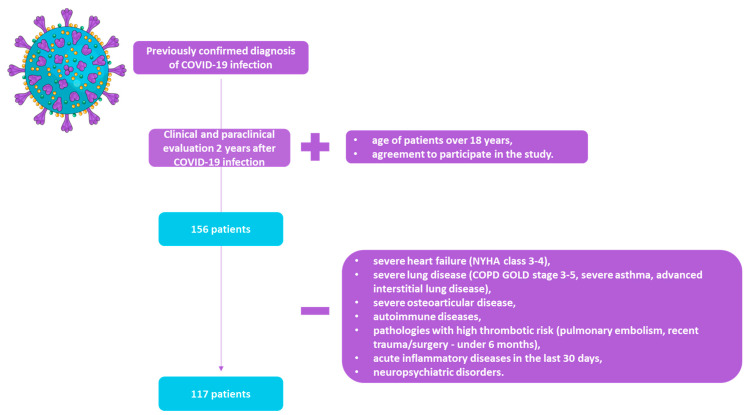
Flow chart of the studied group (COVID-19: coronavirus disease 19; NYHA: New York Heart Association; COPD: chronic obstructive pulmonary disease).

We conducted a retrospective, descriptive study on 156 consecutive patients previously diagnosed with COVID-19 (defined as a positive real-time reverse transcription PCR (RT-PCR) test in nasopharyngeal swabs [22]) and admitted to the Pulmonary Rehabilitation Clinic, Iasi Clinical Rehabilitation Hospital between July 2022 and July 2023 for clinical, biological, and functional reassessment, as well as a supervised respiratory rehabilitation program, at least two years after infection. Due to incomplete medical data (biological and functional parameters), 39 patients were eliminated from the initial study group, resulting in 117 patients in the final group (Figure 1). 

Inclusion criteria were a definite diagnosis of COVID-19 infection at least two years prior to evaluation and the age of patients being over 18 years. Exclusion criteria were patients with co-existent severe chronic respiratory and cardiac diseases as the most probable cause of their symptoms and functional impairment, such as heart failure New York Heart Association [NYHA] class III-IV, chronic obstructive pulmonary disease [COPD] GOLD stage 3–4, severe asthma, or idiopathic pulmonary fibrosis, as well as pathologies severely influencing the thrombotic risk and inflammatory biologic profile (pulmonary thromboembolism, acute myocardial infarction, severe stroke, trauma, surgery in the last six months, acute inflammatory diseases in the last 30 days prior to hospitalization, known major autoimmune systemic diseases, and active or late-stage cancers). Patients with severe osteoarticular impairment and those with major neuropsychiatric disorders were also excluded due to their inability to perform the functional testing, resulting in missing or unreliable data.

We defined two groups according to the presence (N = 49) or absence (N = 68) of documented pulmonary fibrosis; the presence of pulmonary fibrosis was documented by high-resolution computerized tomography 1–3 months after the initial diagnosis of COVID-19 under the European Respiratory Society recommendations [23]. Between groups, there were no statistical differences regarding gender, mean age, or area of residence. We stratify the study group according to the presence of pulmonary fibrosis to compare the long-term evolution of those with imaging-altered lung structure (fibrosis) to those without significant imagistic pulmonary impairment. We chose fibrosis as the most significant post-acute respiratory sequelae of COVID-19 infection, with a possible long-term impact on patients’ respiratory and general state of health. We differentiated post-COVID-19 fibrosis from IPF post-factum at the moment of admission to our hospital based mainly on several clinical aspects: no history of idiopathic pulmonary fibrosis previous to COVID-19 infection or in the two-year interval post-infection; the absence of suggestive clinical elements such as clubbing, myalgias, unexplained weight loss, or low-grade fever; and good results of pulmonary function tests in the fibrosis group (low score of dyspnea on the Borg scale, normal SpO_2_ aa post-6-minute walk test, normal forced vital capacity [FVC] (%), and mild-to-moderate reduction of diffusing capacity of the lungs for carbon monoxide [DLCO]).

### 2.2. Measurements

We included in our study a variety of demographic, anthropometric, biological, and functional parameters assessed in all patients included in the study. We thoroughly evaluated all patients included in the study (in the time interval of July 2022–July 2023) at the time of enrollment, taking into account additional demographic, anthropometric, clinical-paraclinical parameters, and previously documented comorbidities.

#### 2.2.1. General Data, Past Medical History, and Symptoms

Data on demographics, past medical history, alcohol and cigarette consumption, and chronic use of medications were included in the medical records. Associated comorbidities (e.g., hypertension, chronic obstructive lung disease, diabetes mellitus, chronic kidney disease, and obstructive sleep apnea) previously diagnosed were accepted based on medical documents certifying the accomplishment of European and international current guidelines criteria [23,24,25,26,27,28].

Body mass index (BMI) was calculated as the ratio of weight (kg) to height^2^ (m^2^). A calibrated medical scale was used to determine the participants’ body weight by international standards. 

Dyspnea, as a subjective complaint, was assessed through the modified Medical Research Council scale (mMRC) and the Borg scale [22].

Long COVID was defined according to guidelines [29] as signs and symptoms that appeared during or after a COVID-19 infection and persisted for more than 12 weeks without being explained by another diagnosis. 

#### 2.2.2. Laboratory Data

In terms of biological parameters, complete blood count, lipid profile (total cholesterol, low-density lipoprotein cholesterol [LDL], high-density lipoprotein cholesterol [HDL], and triglycerides), carbohydrate profile (serum fasting glucose and glycosylated hemoglobin), renal function (serum creatinine, urea, creatinine clearance, and uric acid), and proinflammatory, prothrombotic status (C-reactive protein [CRP], erythrocyte sedimentation rate [ESR], ferritin, and D-dimers) were assessed in all patients included in the study in the first day of hospital admission for long-term reassessment. In addition, liver enzymes (alanine transaminase [ALT], aspartate transaminase [AST], gamma-glutamyl transferase [GGT], lactate dehydrogenase, and bilirubin) completed the biological picture. The reference interval of our laboratory for D-dimers value was between 1 and 255 ng/mL; any CRP value above 1 mg/dL was considered elevated.

#### 2.2.3. Pulmonary Function Tests (Spirometry, Body Plethysmography) 

All patients carried out pulmonary function tests using Quark PFT and Q-Box^®^ (Cosmed, Rome, Italy), a variable-body plethysmograph with hardware and software for spirometry and lung volumes, airway resistance, and DLCO. The measurements were performed by an experienced examiner, with at least three acceptable measurements/patient, according to the current guidelines of professional societies [30,31,32]. 

Significant spirometry parameters were considered: forced expiratory volume in the first second [FEV1], FVC, FEV1/FVC ratio, and the maximal expiratory flow at 50% of the FVC [MEF50]. All spirometry outcomes were reported at body temperature, ambient barometric pressure, saturated with water vapour [BTPS], and patients’ birth sex, ethnicity, age, height, and weight were recorded for calculating predicting values. Normal values were considered above 80% of the predicted values for FEV1, FVC, and MEF50 and above 70% for the FEV1/FVC ratio [30,32].

Whole-body plethysmography was performed on the same Cosmed Q-Box^®^ platform in standardized conditions, and the parameters of interest were lung volumes, including residual volume [RV], total lung capacity [TLC], and functional reserve capacity [FRC], as well as specific airways resistance [sRaw]. Normal values for lung volumes were between 80 and 120% of predicted values.

Spirometry was interpreted in conjunction with body plethysmography, defining the following ventilatory defects [30,31,32]:

– Obstructive ventilatory pattern if FEV1/FVC ratio is less than 70%, FEV1 is less than 80%, and hyperinflation if increased RV and TLC;
– Restrictive pattern if normal FEV1/FVC ratio (<70%) and a reduction in FVC and TLC under 80% of predicted values;
– Mixed pattern when both FEV1/FVC ratio and TLC, as well FVC, are decreased below predicted values.

The diffusing lung capacity [DLCO] was determined using the single-breath carbon monoxide gas transfer technique, which was adjusted for alveolar ventilation and hemoglobin content [DLCO corr]. Impaired diffusion through the alveolar-capillary membrane was defined as DLCO less than 80% of the predicted value [30,33].

#### 2.2.4. Six-Minute Walk Test

All patients enrolled in the study underwent the 6-minute walk test [6MWT] to assess exercise capacity, according to ATS/ERS Guidelines [32,34]. Prior to the start of the test, waist, weight, and previously administered medication (type, dose) were assessed. A 30 m flat corridor was used, and the number of laps performed (complete laps and partial final lap) was quantified. Test duration, heart rate, blood pressure, dyspnea (quantified using the Borg scale), and SpO_2_ were quantified before and after the test. The main symptoms monitored during the test were angina pectoris, dizziness, and the presence of lower limb pain at different levels (hip, calf, leg). The predicted walking distance, according to gender, resulted from two equations [34] as follows:

- Male gender: 867 − [5.71 × age] + [1.03 × height (cm)] (m);
- Female gender: 525 − [2.86 × age] + [2.71 × height (cm)] − [6.22 × BMI] (m).

#### 2.2.5. Other Data

Blood pressure measurements [BP] were performed according to ESC Guidelines for Hypertension [23]. Electrocardiograms (12-channels) [ECG] were recorded using Beneheart R12^®^ (Mindray, Shenzhen, China) electrocardiograph; the device automatically calculated heart rate [HR] (bpm), QRS duration (ms), and QT interval corrected [QTc] (ms).

### 2.3. Statistical Analysis

We used the Statistical Package for the Social Science (SPSS) statistics software (version 26 for Windows, SPSS Inc., Chicago, IL, USA) to analyze the abovementioned parameters. The results were reported as mean ± standard deviation (SD) for metric variables or frequency and percentages for categorical parameters. We tested the normal distribution of the data using the Kolmogorov–Smirnov test, and then we applied an independent *t*-test to determine the significance of the differences between the groups (men vs. women) for all normally distributed continuous variables and the Mann–Whitney *U* test for those not respecting the normal distribution law; this also applied to the categorical variables. Pearson (for continuous, normally distributed variables) and Spearman (for categorical variables) correlation coefficients were used to assess the association between the studied variables. Receiver operating characteristic (ROC) analysis and univariate and multivariate analyses were performed to identify the predictive value of increased D-dimers in patients with altered capillary diffusion. A *p*-value of ≤0.05 was considered to be statistically significant.

### 2.4. Ethics

The study was approved by the Ethics Committee of the Iasi Clinical Rehabilitation Hospital and was conducted according to the Helsinki Declaration. All patients had signed an informed consent in the medical record which declared their general and medical data could be used for research purposes.

## 3. Results

We analyzed a cohort of 117 patients (69.23% females, mean age of 65.74 ± 10.19 years old) diagnosed two years ago with COVID-19 who presented to our clinic to assess respiratory functional status and respiratory recovery. Patients were divided into two groups according to the presence or absence of pulmonary fibrosis: patients with associated pulmonary fibrosis (*n* = 49, group 2) and patients without residual pulmonary involvement (*n* = 68, group 1). We analyzed a variety of clinical and paraclinical parameters presented in Table 1.

Regarding anthropometric parameters, the average BMI value (31.42 ± 5.71 kg/m^2^) was above the upper limit of normal range in the whole group. It was slightly higher among patients with pulmonary fibrosis but without statistical significance (31.15 ± 5.11 vs. 32.16 ± 6.27, *p* = 0.975). No statistically significant differences were reported according to the presence or absence of pulmonary fibrosis post-COVID-19 among demographic or anthropometric parameters. 

ECG parameters revealed that mean HR was significantly higher among patients with pulmonary fibrosis (*p* < 0.05), as was the mean value of the QTc interval (297.12 ± 158.05 ms vs. 362.11 ± 126.45 ms, *p* < 0.05).

Associated comorbidities were diverse, the most common being hypertension (66.67%), chronic ischemic heart disease (28.21%), asthma (mild forms, 17.95%), and mild-to-moderate heart failure (17.97%). Statistical analysis revealed that among those with radiological changes of lung fibrosis, a higher percentage of hypertension (64.71% vs. 69.39%, *p* = 0.596), mild COPD (13.24% vs. 26.53%, *p* = 0.069), chronic ischemic heart disease (25% vs. 32.65%, *p* = 0.596) and heart failure (14.71% vs. 20.41%) were found, without associated statistical significance. 

Regarding biological parameters, no significant differences in lipid parameters were reported between the two groups. However, non-significant higher mean serum levels of LDL-cholesterol were reported in patients with COVID-19 and pulmonary fibrosis (*p* = 0.642). From the liver function tests, only AST has a statistical significance (*p* < 0.05), the mean value being higher in fibrosis group.

In terms of inflammatory markers, patients with pulmonary fibrosis were associated with a significantly higher mean CRP value than patients in the first group (*p* = 0.048). The persistence of a notable proinflammatory status at a long-term distance from the acute episode was also revealed by higher mean WBC (7.45 ± 7.86/mm^3^ vs. 9.18 ± 17.24/mm^3^, *p* = 0.053), neutrophil values (4.68 ± 7.88/mm^3^ vs. 9.07 ± 17.44/mm^3^, *p* < 0.05), and mean MPV (7.22 ± 0.93 vs. 10.25 ± 0.86 fL, *p* < 0.05) in the fibrosis group. The same group seemed to preserve significantly higher levels of D-dimer (*p* < 0.05) and serum lactate dehydrogenase (*p* < 0.05) but not ferritin (*p* = 0.470). Lower hemoglobin levels were also noted in patients with associated pulmonary fibrosis vs. non-fibrotic patients, without significance (14.70 ± 16.57 vs. 13.01 ± 1.52 g/dL, *p* = 0.395) (Table 1). No patient has experienced a thromboembolic event within two years of COVID-19 diagnosis.

The results of functional testing are shown in the table above. In terms of spirometry, mean percentage values, expressed as % of predicted values, of FEV1 (*p* = 0.545), FVC (*p* = 0.700), and MEF50 (*p* = 0.831) were slightly lower in patients in the second group, but without statistical significance. Similarly, in the case of body plethysmography, the values of pulmonary volumes recorded at a two-year distance from COVID-19 showed no apparent ventilatory dysfunction. 

The 6-minute walk test was performed in all patients to assess exercise capacity objectively, long-term after the acute episode of COVID-19. The mean values of walking distance (365.74 ± 142.29 m vs. 366.51 ± 133.36 m, *p* = 0.847), SpO_2_ post-test (96.28 ± 1.80% vs. 96.05 ± 1.52%, *p* = 0.238), and the product of the two parameters mentioned above (36,332.44 ± 13,612.37 vs. 33,755.23 ± 12,855.12, *p* = 0.307) were approximately equal in the two groups. 

At the end of the test, the level of subjectively felt dyspnea was assessed using the Borg scale; the mean score was similar between the two groups and in the mid-lower range of the 10-point scale (2.77 ± 0.86 vs. 3.04 ± 2.02, *p* = 0.275). Furthermore, in patients with pulmonary fibrosis, there was a moderate positive and significant correlation between DLCO corr (% from the predicted value) and the distance covered in the 6-minute walk test (r = 0.370, *p* < 0.05) but not with post-test SpO_2_ (r = 0.286, *p* = 0.132). A stronger positive correlation was found among patients without pulmonary fibrosis (r = 0.459, *p* < 0.01). 

**Figure 2:**
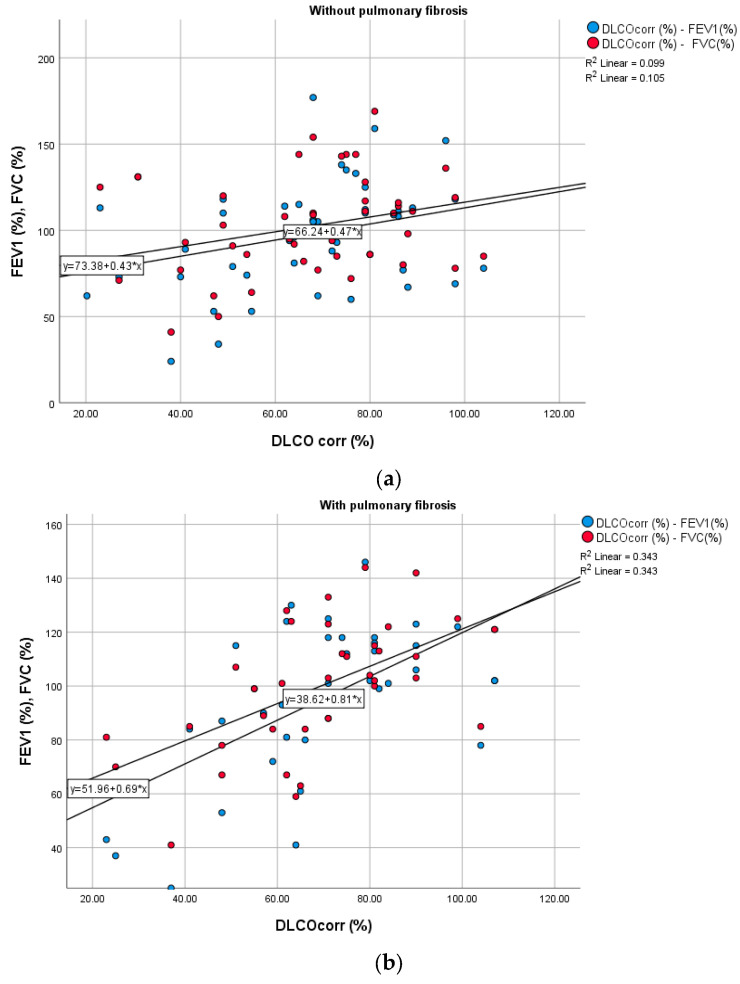
Correlation between DLCO corr (%), FEV1 (%), and FVC (%) in patients post-COVID-19, without pulmonary fibrosis (**a**) and with pulmonary fibrosis (**b**). (DLCO: diffusing capacity of lung for carbon monoxide; FEV1: forced expiratory volume in 1 s; FVC: forced vital capacity).

**Figure 3:**
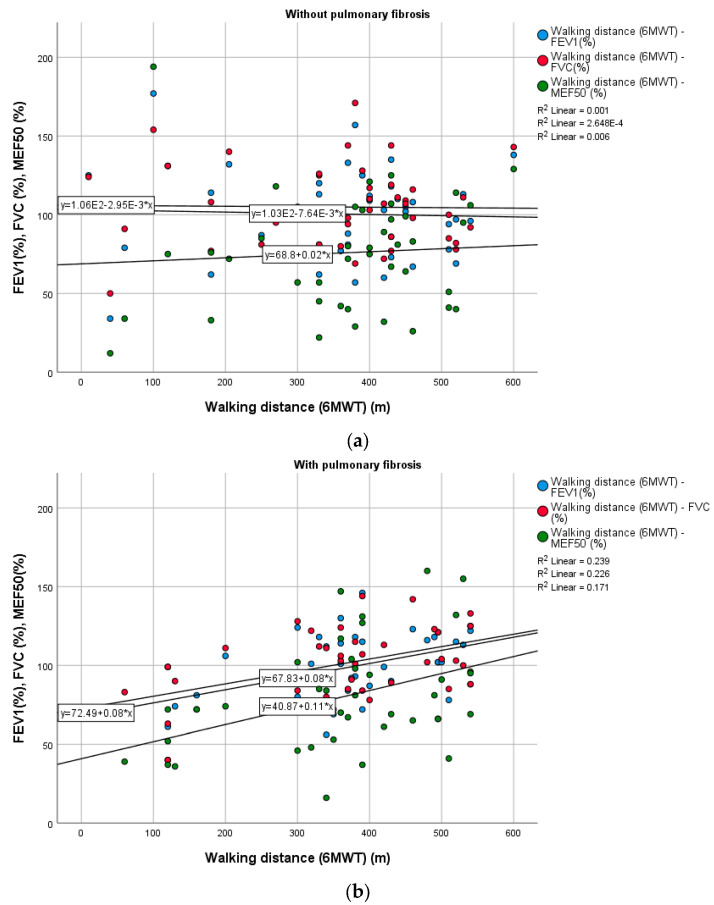
Correlation between the walking distance (m), FEV1 (%), and FVC (%) in patients post-COVID-19, without pulmonary fibrosis (**a**) and with pulmonary fibrosis (**b**). (6MWT: 6-minute walking test; FEV1: forced expiratory volume in 1 s; FVC: forced vital capacity).

Table 2 and Figure 2 and Figure 3 display the correlations between the parameters of pulmonary function tests (spirometry and body plethysmography) and walking distance, a surrogate marker of exercise capacity. In the group of patients with pulmonary fibrosis, walking distance correlated positively and significantly with FEV1 (*p* = 0.006), FVC (*p* = 0.018), and MEF50 (*p* = 0.029) (Figure 2). In the group of patients post-COVID-19 without pulmonary fibrosis, DLCO corr values (%) and distance covered in the 6-minute walk test significantly correlated with inflammatory marker CRP. Moreover, DLCO corr (% from the predicted value) measured in patients with pulmonary fibrosis was strongly negatively correlated with D-dimer values (r = −0.621; *p =* 0.038).

**Figure 4:**
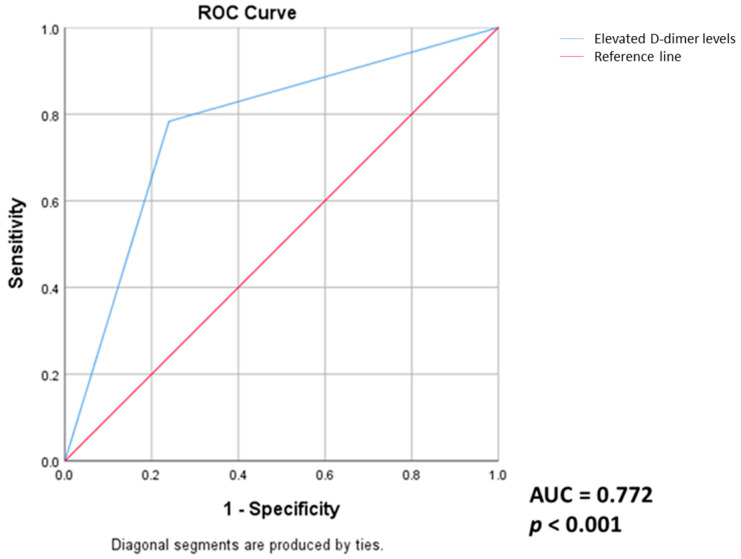
ROC analysis. Elevated D-dimer levels could act as a predictor for long-term persistence of decreased DLCO values in patients post-COVID-19 (AUC: area under the curve).

Based on literature data reporting an association between elevated D-dimer values and reduced DLCO values and our findings of a strong significant correlation between D-dimers and DLCO corrected for hemoglobin, we tested further the link between the persistence of elevated D-dimer values and reduced DLCO values (less than 80% of predicted values) two years after COVID-19. Receiver operating characteristic (ROC) analysis was performed to calculate the area under the curve for the persistence of elevated D-dimer values in order to determine if this parameter could act as a predictor associated with the long-term persistence of decreased values of DLCO in patients post-COVID-19 (area under the curve of 0.772 and *p* < 0.001) (Figure 4).

## 4. Discussion

The present research considers the long-term impact of COVID-19 infection two years after infection through evaluating biological and functional parameters with therapeutic and prognostic implications. 

Demographic factors contribute to the persistence of long-COVID-associated symptoms, with Debski et al. [35] demonstrating that female gender is a risk factor and increasing BMI by every 1 kg/m^2^ is accompanied by a 3% relative risk increase (odds ratio 1.031). Similar results were reported in our research, with the proportion of women over 65% in both groups. Additionally, the mean BMI value was over 31 kg/m^2^ in both groups, 1 kg/m^2^ higher among patients with pulmonary fibrosis, consistent with the 3% relative risk increase for the persistent symptoms [35]. In a similar clinical study, Bai et al. [36] showed by multivariate statistical analysis that female gender is an independent risk factor for the development of long COVID, the risk being 3.3 times higher compared to male patients (*p* < 0.0001).

Florencio and Fernández-de-las-Peñas [37] emphasized in patients with long COVID the persistence of an inflammatory status after the acute infectious stage, maintained and stimulated by individual factors, including obesity, which brings into question the potential use of anti-inflammatory therapeutic agents in this population.

Comorbidities modulate the long-term prognosis of patients with a history of COVID-19 through the severity of symptoms and impact on morbidity and mortality. In a prospective study, Pérez-González et al. [38] demonstrated that COPD (*p* = 0.018), female gender (*p* < 0.001), and smoking (*p* = 0.049) are risk factors for the development of long COVID. In our study, the percentage of patients with COPD was double in the second group (with pulmonary fibrosis), similar to smoking, but with no associated significance and a low number of such cases.

Adegunsoye et al. [39] investigated the role of medication, such as amiodarone, corticosteroids, rituximab or chemotherapy, in modifying the risk of pulmonary fibrosis after COVID-19. However, none of our patients were subject to this type of pharmacotherapy, meaning the development of pulmonary fibrosis resulted from the infection.

Patients with long-COVID-associated cardiac autonomic dysfunction (secondary to autonomic nervous system disorders) manifested as increased heart rate and decreased heart rate variability, which leads to increased cardiovascular morbidity and mortality [40]. Clinical studies conducted so far have hypothesized a connection between inflammatory markers (CRP, leukocytes) and increased heart rate, but further studies are needed to confirm this. In our study, the mean heart rate and QTc interval were significantly higher among patients with pulmonary fibrosis. Furthermore, we found a moderate negative correlation between QTc interval and DLCO corr in the pulmonary fibrosis group, along with a higher resting heart rate, raising the question of long-term impairment of the autonomic nervous system in those patients, with the prominence of sympathetic drive and increased cardiovascular risk.

Pasini et al. [41] evaluated the metabolic profile in a cohort of 75 patients with long COVID. They observed elevated concentrations of ferritin, D-dimer, ESR, hs-CRP and LDH. The same group of investigators showed decreased serum levels of the above parameters with the increasing time intervals from COVID-19. However, they maintained mean serum levels above the upper limit, supporting the proinflammatory and prothrombotic status hypothesis. The evaluation performed two months after the remission of the acute episode showed the persistence of low hemoglobin values, which we also found after a much more extended follow-up period (2 years). Patients with long COVID are also associated with various hematological disorders [42]; erythrocyte and derived parameter modifications are due to deformability changes and structural alterations observed eight months after the acute episode [43]. In our study, the mean values of platelets (*p* < 0.05) and hemoglobin (*p* < 0.05) were lower in the pulmonary fibrosis group. In comparison, the values of leukocytes (*p* = 0.053), neutrophils (*p* < 0.05), and MPV (*p* < 0.05) were significantly higher, supporting the observation of a persistent chronic inflammatory status even two years after acute COVID-19 infection.

The role of platelets in the occurrence of COVID-19-associated thromboembolic events has been evaluated in multiple studies published in the literature, but the pathophysiological mechanisms still need to be fully elucidated. Shape changes, platelet-mediated immunoregulation [44], or changes associated with the activator phenotype are some pathophysiological hypotheses developed so far [45,46,47]. Mean platelet volume [MPV] is a prognostic marker frequently used in association with cardiovascular risk, with higher values associated with prothrombotic and proinflammatory status [48], this biological finding being confirmed in our study as well for the group with pulmonary fibrosis, long-term after COVID-19.

Demircioglu et al. [49] proposed a new staging system for long-COVID-associated pulmonary fibrosis, the main clinical parameters with a negative prognostic role and correlated with the fibrosis score being elevated lactate dehydrogenase values, lymphocyte count, elevated hs-CRP value, and duration of hospitalization. In our study, we demonstrated that in the group of patients with fibrosis, patients have associated higher serum values of the LDH, CRP, and lymphocyte count. 

Ceban et al. [50] conducted a meta-analysis of 68 clinical trials including patients with COVID-19 evaluated at least 12 weeks after remission of the acute episode. They found persistence of fatigue (*p* < 0.001) and functional decline (*p* < 0.001) after resolution of acute symptoms associated with persistent inflammatory status. Several investigators have reported elevated CRP values in up to 25% of patients in various analyses of large cohorts of patients, similar to elevated serum D-dimer values, supporting the maintenance of a proinflammatory and prothrombotic status long after the acute episode has subsided [51]. In our study, two years after COVID-19, mean D-dimer values were within the normal range but significantly higher among patients with associated pulmonary fibrosis (*p* = 0.018) and strongly and negatively correlated with functional impairment reflected in the values of DLCO corr (r = −0.621; *p* < 0.05).

Sonnweber et al. [52] evaluated 145 patients with COVID-19 at 60 and 100 days after remission of the acute episode. They found persistence of respiratory distress (judged as decreased DLCO and static or dynamic lung volumes) but with moderate and significant improvement over time (*p* < 0.05 for all parameters). Regarding the evolution of biological markers, the same group of researchers reported the persistence of elevated CRP (12% of patients), D-dimer (27%), and serum ferritin (17%) values 100 days after the acute event. Our research demonstrated the persistence of a chronic inflammatory status even two years after the acute event by identifying above-normal mean CRP, ESR, WBC, and neutrophil values. Mean ferritin values were within the normal range in both groups. However, we interpreted this because ferritin is an acute inflammatory marker and high values are not extended two years after the acute COVID-19 episode. Moreover, using exclusion criteria, we have eliminated patients with various acute inflammatory pathologies that could have influenced the results of our research.

The persistence of elevated serum D-dimer levels has been observed in assessments performed after remission of the acute episode of COVID-19. It has been reported in numerous clinical studies to date. The group of investigators led by Venturelli et al. [53] analyzed a cohort of 767 patients and observed that 38% of patients had elevated D-dimer values 81 days after COVID-19 infection. George et al. [54] showed the persistence of an inflammatory phenotype associated with neutrophilia in patients with COVID-19 evaluated one year after the acute episode, particularly among patients with respiratory symptoms. We also found that in those patients with parenchymal scarring a short time after acute infection (1–3 months), there is a persistent mild elevation of D-dimers level, compared to those without pulmonary involvement at the time, even long-term after the acute episode (two years or more). We considered this finding significant for an ongoing subclinical activation of coagulation and fibrinolytic system in these individuals as we excluded all patients with known alternative causes of D-dimer elevation from our study from the beginning.

Another interesting issue is lipid metabolism, which is highly active in the lungs, with pathophysiological studies demonstrating the presence of a significant percentage of lipids in the alveolar surfactant [55]. Our study did not show statistically significant differences in lipid profile parameters (*p* > 0.05 for all parameters). Among all lipid profile parameters, the assessment performed two years after COVID-19 infection showed higher mean serum values in patients with associated pulmonary fibrosis, as presented in the Results section. In a similar, recently published clinical study, Durrington obtained similar results for LDL-cholesterol, suggesting periodic evaluation of the lipid profile for cardiovascular risk assessment and the need to initiate lipid-lowering treatment [56].

Several clinical studies have demonstrated the role of cholesterol in modulating the inflammatory and remodelling processes in the lungs [57,58]. Although alterations in metabolic pathways related to fatty acids are linked particularly with pulmonary idiopathic fibrosis [57], systemic and pulmonary lipid metabolism could also contribute to the tissue scarring process in post-COVID-19 pulmonary fibrosis. Moreover, apolipoprotein A-I has attracted researchers’ attention because, when dysfunctional or low-level, it favors the increased formation of foam cells, as noted in the lungs of patients with different types of pulmonary fibrosis [59]. Unfortunately, we did not determine the level of apolipoprotein A-I along with the lipid profile of post-COVID patients, but this could be a promising research direction. 

Regarding the functional respiratory status after COVID-19, Suppini et al. [60] analyzed a cohort of 140 patients previously diagnosed with acute infection. They observed at the 1-year follow-up an improvement in DLCO (61.7 ± 21.5 vs. 68.9 ± 19.9) and TLC values (77.9 ± 21.0 vs. 83.8 ± 19.0) (*p* < 0.05 for both) regardless of associated severity. The same group of investigators showed improvement in pulmonary dysfunction as measured by improvement in mean FEV1 (*p* = 0.054) and FVC (*p* = 0.020). These aspects suggest at least partial reversibility of functional impairment secondary to acute infection in the long run, consistent with the findings in our cohort. 

Our study had several limitations, the first being represented by the heterogeneity of the study group, but we also must consider that COVID-19 affected the heterogeneous real-life population. Another limit is the small size of the groups, but this was conceived as a pilot study in order to see if any significant biological or functional impairment remains in various individuals long-term after the acute episode of infection, with a particular interest in prothrombotic profile and functional impairment affecting the quality of life and normal functioning of individuals. We also consider as a limitation the evaluation of exercise capacity through the 6MWT instead of the gold standard of the cardiopulmonary exercise test. However, the latter was performed only shortly after COVID-19 and later only in highly affected or symptomatic patients for prognostic and therapeutic reasons. The same reason is behind another significant gap of our study: the absence of a systematic HR-CT pulmonary scan at the moment of readmission in the hospital, two or more years after the acute episode of infection, for the long-term assessment of the COVID-19 patients, due to financial burden. 

To better characterize the persistence of inflammatory/prothrombotic status, it would have been of significant interest to compare the biological parameters at baseline (acute COVID-19 infection) with those at reassessment two or more years later and between the two groups. Moreover, systematic baseline data regarding the severity of initial infection, such as mechanical ventilation, duration of hospitalization, and admission to ICU, would be beneficial in better characterizing the risk profile of the two groups—fibrosis and non-fibrosis—at baseline and their long-term evolution. A significant limitation in realizing such a prospective analysis was the lack of detailed medical data regarding the acute episode as we did not have access to the entire medical record of every patient; the majority of the patients were admitted to other medical units during the acute episode of COVID-19 and to our rehabilitation hospital only for long-term reassessment and pulmonary rehabilitation program. Consequently, we must exclude such information on acute infection due to the numerous data gaps on all included patients and use only the summary information on the discharge documents and the imagistic results. 

## 5. Conclusions

In this pilot study, we investigated the long-term impact of COVID-19 infection, mainly focusing on proinflammatory, prothrombotic markers and functional status at least two years after the acute episode. We have compared patients with COVID-19-related pulmonary fibrosis versus patients without parenchymal lung involvement. Both patient groups exhibited no significant persistent dyspnea, substantial impairment in pulmonary function tests, or diminished exercise capacity. However, the exploration of biomarkers revealed higher levels of D-dimers, mean platelet volume (MPV), lactate dehydrogenase (LDH), C-reactive protein (CRP), and neutrophils in patients with COVID-19-related fibrosis, probably reflecting a persistent subclinical low-grade proinflammatory and prothrombotic status in these individuals. Our study identified a strong negative correlation between D-dimers and diffusing capacity for carbon monoxide (DLCO) values. The persistence of elevated D-dimer levels could act as a predictive factor for long-term impaired DLCO, underscoring the need for further investigations to unravel its therapeutic and prognostic implications.

Despite the inherent limitations of our small-scale pilot study, a more extensive investigation will yield more robust data, contributing to deepening our understanding of the long-term implications of the recent and dramatic COVID-19 pandemic.

## Figures and Tables

**Table 1:** Demographics, anthropometric parameters, vitals and biological data.

Parameter	Total Group (*n* = 117)	Without Pulmonary Fibrosis (*n* = 68)	With Pulmonary Fibrosis (*n* = 49)	*p*
**Demographics**				
Age	65.74 ± 10.19	65.04 ± 9.07	66.36 ± 11.57	0.270
Gender (females)	81 (69.23%)	49 (72.06%)	32 (65.31%)	0.435
Area of residence (urban)	70 (59.82%)	41 (60.29%)	29 (59.18%)	0.904
**Anthropometric data**				
Height, m	1.65 ± 0.28	1.61 ± 0.09	1.71 ± 0.43	0.027
Weight, kg	82.21 ± 15.64	82.02 ± 13.91	82.96 ± 18.06	0.566
BMI, kg/m^2^	31.42 ± 5.71	31.15 ± 5.11	32.16 ± 6.27	0.975
**Comorbidities**				
COPD	22 (18.80%)	9 (13.24%)	13 (26.53%)	0.069
OSA	14 (11.97%)	9 (13.24%)	5 (10.20%)	0.618
Asthma	21 (17.95%)	13 (19.12%)	8 (16.33%)	0.698
Hypertension	78 (66.67%)	44 (64.71%)	34 (69.39%)	0.596
Chronic coronary syndromes	33 (28.21%)	17 (25.00%)	16 (32.65%)	0.364
Arrhythmias/atrio-ventricular conduction disorders	26 (22.22%)	11 (16.18%)	15 (30.61%)	0.064
Stroke	5 (4.27%)	4 (5.88%)	1 (2.04%)	0.311
Heart failure	20 (17.97%)	10 (14.71%)	10 (20.41%)	0.419
Type 2 diabetes mellitus	40 (34.19%)	28 (41.18%)	12 (24.49%)	0.060
Chronic kidney disease	10 (8.62%)	3 (4.41%)	7 (14.58%)	0.055
Neoplasia	5 (4.27%)	2 (2.94%)	3 (6.12%)	0.401
Active smoking	11 (9.40%)	5 (7.35%)	6 (12.24%)	0.371
Alcohol consumption	17 (14.5%)	11 (16.18%)	6 (12.24%)	0.552
**Biological data**				
CRP, mg/dL	1.32 ± 2.02	0.98 ± 0.56	1.82 ± 3.07	0.048
ESR, mm/h	21.56 ± 14.51	20.47 ± 12.25	23.32 ± 17.46	0.737
Hemoglobin, g/dL	13.98 ± 12.66	14.70 ± 16.57	13.01 ± 1.52	0.395
Hematocrit, %	38.95 ± 12.66	38.83 ± 3.33	39.88 ± 4.83	0.311
MCV	92.83 ± 5.22	92.44 ± 5.26	93.43 ± 5.27	0.509
Platelets (×10^3^/mL)	263.21 ± 68.56	266.68 ± 62.48	242.66 ± 75.13	0.047
MPV, fL	8.93 ± 0.89	7.22 ± 0.93	10.25 ± 0.86	0.038
WBC (×10^3^/mm^3^)	8.10 ± 12.46	7.45 ± 7.86	9.18 ± 17.24	0.053
Neutrophils (×10^3^/mm^3^)	6.40 ± 12.70	4.68 ± 7.88	9.07 ± 17.44	0.032
Lymphocytes (×10^3^/mm^3^)	3.15 ± 5.54	2.88 ± 4.90	3.61 ± 6.49	0.856
Eosinophils (×10^3^/mm^3^)	0.28 ± 0.42	0.26 ± 0.37	0.31 ± 0.48	0.575
D-dimers, mg/L	264.43 ± 130.37	245.57 ± 78.19	283.29 ± 160.32	0.018
Lactate dehydrogenase, UI/L	321.13 ± 117.53	296.19 ± 73.76	349.63 ± 151.33	0.039
Ferritin	109.77 ± 77.30	101.65 ± 75.40	119.71 ± 80.60	0.470
Total cholesterol, mg/dL	204.03 ± 46.30	200.41 ± 44.90	208.62 ± 49.18	0.344
LDL-cholesterol, mg/dL	125.56 ± 41.03	123.03 ± 38.24	130.47 ± 46.04	0.642
HDL-cholesterol, mg/dL	65.16 ± 15.57	63.27 ± 15.84	66.12 ± 14.68	0.205
Triglycerides, mg/dL	137.12 ± 60.29	139.15 ± 57.67	136.99 ± 64.28	0.460
ALT, UI/L	22.57 ± 12.56	21.53 ± 11.46	24.15 ± 14.13	0.611
AST, UI/L	20.47 ± 8.34	19.28 ± 7.79	21.91 ± 8.70	0.023
Total bilirubin, mg/dL	0.72 ± 0.91	0.77 ± 1.14	0.61 ± 0.26	0.700
Direct bilirubin, mg/dL	0.46 ± 2.59	0.68 ± 3.51	0.18 ± 0.09	0.732
Alkaline phosphatase, UI/L	85.88 ± 23.43	89.10 ± 25.68	81.30 ± 19.85	0.606
GGT, UI/L	40.93 ± 60.13	37.73 ± 67.02	46.39 ± 50.29	0.219
Uric acid, mg/dL	4.70 ± 1.52	4.50 ± 1.25	4.97 ± 1.82	0.180
Fasting glucose, mg/dL	114.24 ± 28.00	116.01 ± 32.49	112.15 ± 20.61	0.895
Glycosylated hemoglobin	6.93 ± 1.63	7 ± 1.62	6.81 ± 1.72	0.714
**Vitals, ECG derived parameters**				
HR, bpm	70.24 ± 13.88	68.26 ± 12.23	73.37 ± 15.90	0.033
QT interval corrected, ms	326.13 ± 148.20	297.12 ± 158.05	362.11 ± 126.45	0.018
QRS duration, ms	191.48 ± 147.77	211.48 ± 157.22	164.55 ± 130.11	0.705
**Spirometry**				
FEV1, (%)	95.83 ± 29.36	97.33 ± 29.39	92.53 ± 29.47	0.545
FVC, (%)	100.38 ± 26.75	101.25 ± 25.44	98 ± 28.67	0.700
FEV1/FVC	78.68 ± 12.29	78.93 ± 11.48	78.30 ± 13.73	0.747
MEF50, (%)	74.09 ± 38.60	74.35 ± 38.27	73.33 ± 40.28	0.831
**Body Plethysmography**				
DLCO corr (%)	67.39 ± 20.66	69.53 ± 18.82	65.70 ± 22.02	0.311
DLCO corr (ml)	15.59 ± 5.55	16.08 ± 5.36	15.23 ± 5.74	0.448
Body CRF	96.30 ± 29.58	95.12 ± 27.63	98.29 ± 32.63	0.827
Body CPT	91.11 ± 22.49	88.02 ± 21.43	95.21 ± 23.76	0.125
Body specific airway resistance	7.24 ± 4.70	6.93 ± 4.44	7.66 ± 5.13	0.514
**6-minute walk test**				
Distance, m	366.09 ± 137.45	365.74 ± 142.29	366.51 ± 133.36	0.847
SpO_2_ post-test, %	96.17 ± 1.67	96.28 ± 1.80	96.05 ± 1.52	0.238
Distance–saturation product	35,271.24 ± 13,289.31	36,332.44 ± 13,612.37	33,755.23 ± 12,855.12	0.307
Dyspnea (Borg scale)	2.89 ± 1.46	2.77 ± 0.86	3.04 ± 2.02	0.275

BMI: body mass index; ECG: electrocardiogram; HR: heart rate; COPD: chronic obstructive pulmonary disease; OSA: obstructive sleep apnea syndrome; LDL: low-density lipoprotein cholesterol; HDL: high-density lipoprotein cholesterol; ALT: alanine transaminase; AST: aspartate transaminase; GGT: gamma-glutamyl transferase; CRP: C-reactive protein; ESR: erythrocyte sedimentation rate; MCV: mean corpuscular volume; WBC: white blood cells; MPV: mean platelet volume; FEV1: forced expiratory volume in 1 s; FVC: forced vital capacity; MEF50: maximal expiratory flow at 50% of the forced vital capacity; DLCO: diffusing capacity of lung for carbon monoxide.

**Table 2:** Correlations between proinflammatory and prothrombotic markers and functional parameters.

	With Pulmonary Fibrosis				Without Pulmonary Fibrosis			
	DLCO corr		Distance (m)		DLCO corr		Distance (m)	
	r	*p*	r	*p*	r	*p*	r	*p*
D-dimers	−0.621	0.038	−0.213	0.381	−0.419	0.045	−0.128	0.580
LDH	−0.466	0.127	−0.024	0.942	−0.123	0.703	−0.526	0.079
CRP	−0.251	0.046	−0.120	0.474	−0.413	0.003	−0.404	0.006
Platelets	−0.108	0.537	0.095	0.566	−0.184	0.200	−0.037	0.807
MPV	−0.026	0.882	−0.072	0.663	0.040	0.783	−0.017	0.911
Dyspnea (Borg scale)	−0.212	0.228	−0.092	0.598	−0.133	0.378	−0.108	0.478
FEV_1_	0.544	0.001	0.445	0.006	0.293	0.043	−0.046	0.769
FVC	0.623	<0.001	0.387	0.018	0.353	0.014	0	0.997
MEF_50_	0.288	0.094	0.360	0.029	0.326	0.025	0.202	0.193
QTc	−0.339	0.050	−0.152	0.368	0.074	0.625	−0.283	0.059

r: Pearson correlation; DLCO: diffusing capacity of lung for carbon monoxide; LDH: lactate dehydrogenase; CRP: C-reactive protein; MPV: mean platelet volume; FEV_1_: forced expiratory volume in 1 s; FVC: forced vital capacity; MEF_50_: maximal expiratory flow at 50% of the forced vital capacity.

## Data Availability

All the data are available from the corresponding author upon reasonable request.
